# Stand structure influences understory plant diversity through soil factors: three afforestation types of Masson’s pine in the upper Yangtze River, China

**DOI:** 10.3389/fpls.2025.1513038

**Published:** 2025-05-30

**Authors:** Yongqi Xiang, Qian Lyu, Huiqin Yang, Biran Yin, Zhiren Tang, Guirong Hou, Gang Chen, Kuangji Zhao, Yuqin Chen, Chuan Fan, Xianwei Li

**Affiliations:** ^1^ College of Forestry, Sichuan Agricultural University, Chengdu, China; ^2^ National Forestry and Grassland Administration Key Laboratory of Forest Resources Conservation and Ecological Safety on the Upper Reaches of the Yangtze River& Forestry Ecological Engineering in the Upper Reaches of the Yangtze River Key Laboratory of Sichuan Province, Chengdu, China

**Keywords:** diversity, ecological niche, mixed afforestation, *P. massoniana*, stand spatial structure, understory plant community structure

## Abstract

**Purpose:**

In general, mixed forests have the potential to enhance understory plant diversity. However, the effects of stand spatial structure formed by different types of mixed afforestation on understory plants communities are still not clear.

**Methods:**

To answer these questions, we examined the stand spatial structure’s impact on soil (nitrogen, phosphrous, potassium, etc.) and understory plant communities (diversity indexes, ecological niche width and resource overlap of shrub and herb) in three types of *Pinus massoniana* afforestation: a monoculture (MPF), a mixed forest with *Cunninghamia lanceolata* (MCLMF), and a mixed forest with *Liquidambar formosana* (MLMF).

**Results:**

MCLMF substantially increased diversity and ecological niche width for understory shrubs and herbs, steered understory plants toward resource utilization generalism. MLMF enhanced shrub diversity by reducing dominant species ratios. In terms of stand structure, MCLMF significantly increased the opening degree (O), mingling index (M), and competition index (CI), while MLMF decreased CI but increased M. Redundancy analysis indicated that the opening degree explained 52.47% of the variation in shrub diversity and 42.51% in herb diversity, and CI explained 24.57% of the shrub diversity variation. Soil pH, total nitrogen, and available potassium were significantly enhanced after mixed afforestation. The indices O, CI, and M indirectly affect the diversity of understory plants through soil properties, such as temperature, moisture, available nutrients (e.g., nitrogen, phosphorous, and potassium), organic carbon, and pH.

**Conclusions:**

Stand spatial structure significantly shapes understory plant community structure through soil mediation, demonstrating its role in enhancing artificial forest quality and stability in ecologically sensitive areas.

## 1 Introduction

Understory shrub herbaceous vegetation is an essential part of forest ecosystems, playing a significant role in soil and water conservation, promoting nutrient cycling, and maintaining sustainable ecosystem development (Fujii et al., 2017; He, 2020). However, the expansion of artificial forests over recent decades has significantly exacerbated the damaged to forest plant diversity due to monoculture forests (FAO and UNEP, 2020). This issue threatens the sustainable development of forest ecosystems and impacts our living environment. From an ecological promotion perspective, multiple promotion strategies are emerging globally to balance out plant diversity losing (Fu et al., 2015; Wang et al., 2019). Mixed afforestation, as a good choice, can effectively improve soil environment for enhancing stand productivity (Paul et al., 2018) and plant diversity (Fu et al., 2015; Rawlik et al., 2018). However, little attention has been given to stand structure following different mixed afforestation types. In particular, how stand structure influences understory plant diversity and ecological niche potential remains unclear. This knowledge gap may hinder a full understanding of how forest ecosystems regulate understory plant community structure.

As an important part of stand structure, stand spatial structure refers to the distribution pattern of trees and their attributes in space (Hui et al., 2018). Mixed forests essentially involve the coexistence of various tree species, which form diverse stand spatial structures as they grow (Yang et al., 2023). The spatial structures quantitatively reflects the growth status of trees, the level of mixing, the intensity of competition, and the spatial relationship of each neighboring tree in both horizontal and vertical directions (Kershaw et al., 2010), by relying on indicators such as the mingling index, competition, neighborhood comparison, and openness. The stand spatial structure may influences soil physicochemical properties related to the composition of understory plants communities by regulating understory light, water, heat resources through spatial allocation (Luan et al., 2012). For example, studies showed that mingling index M can mitigate soil acidification and increase the available nutrient content, and openness O can increase soil organic matter content (He et al., 2022; Xiang et al., 2024). The spatial structure can also regulate the understory environment, thereby affecting the survival strategies and ecological niche of understory plants (Gadow et al., 2012). Therefore, both the composition structure and resource utilization of the understory plant community are affected by the spatial structure of the upper trees, and these effects can be direct or indirect through environmental factors. Current stand research primarily focuses on nonspatial structures, often based on human-controlled disturbances such as logging (He et al., 2022), with limited studies on stand spatial structure impacts.

Different stand types exhibit varied stand spatial structures, ecosystem functions, environmental regulation abilities, and stability against disturbances. For instance, mixed stands resist pests and diseases better than pure stands (Skovsgaard et al., 2017), and the canopies of broad-leaved species shade the stand light more than coniferous trees (Zhu et al., 2018). Stand spatial structures vary at different developmental stages (forest ages), causing ecosystem function and resource utilization efficiency instability. However, they stabilize their stand structure, ecological function, and resource utilization efficiency as they stand near maturity (Chatterjee et al., 2008; Ouyang et al., 2019; Zhu et al., 2023). Although there have been many studies on stand spatial structure in pure forests or mixed forests (Shi et al., 2016; Wan et al., 2019), mostly focused on the young and middle-aged forests, which leads to the results of these studies on stand spatial structure may only have short reference value. At present, the comparative analysis between different mixed afforestation and pure forest is still lacking after the stand structure is stable, especially in the river along ecosystem.

As the destructive use of natural forests and the large-scale pure forest afforestation, plant diversity decline is rapidly becoming an urgent ecological balance challenge, especially in fragile ecosystems along rivers (Wordley et al., 2017; Chen et al., 2023). For example, in the Amazon Basin, vegetation diversity is being lost, resulting in soil erosion and an increase in greenhouse gases (Soares-Filho et al., 2006). The Yangtze River Basin in China was once plagued by issues such as soil erosion and desertification (Chen et al., 2019). The protection of understory plant diversity is crucial for the stability and healthy development of the ecological environment. Shrubs and herbs are the main components of understory plants and the growth and distribution of understory shrubs and herbaceous are affected by environmental factors such as light, temperature, water, and soil physical and chemical properties (Gao et al., 2019; Liu et al., 2021). Understory plants are sensitive to external disturbances, and can change their species composition and distribution (species diversity) to achieve the optimal ecological niche (Pykälä, 2017; Rowe and Speck, 2005). The spatial structure of tree layer significantly influences the survival conditions of understory plants, particularly the soil environment. However, one system research is still lacking on how stand spatial structure impacts the understory plant community structure and ecological niche change through soil environment after conducting different mixed afforestation especially in fragile ecosystems along rivers.

*Pinus massoniana* is an essential tree species in the upper reaches of the Yangtze River. This species is widely used in artificial forest cultivation (Wang et al., 2022b). However, due to pure forest cultivation and global change, artificial forests of *P. massoniana* may experience problems, such as loss of diversity and decline in soil quality (Wang et al., 2022b). Experiments were conducted on three stand types (monoculture *P. massoniana* forests, *P. massoniana*–*Cunninghamia lanceolata* forests, and *P. massoniana*–*Liquidambar formosana* forests) in the Parallel Ridge Valley area of the upper reaches of the Yangtze River to solve the problem of monoculture planting and diversity declining. From the upper layer to the under layer of the forest, we built a relationship model among the spatial structure of the forest stand, the understory plant community, and the underground soil environment. We systematically studied the source factors that affect the understory plant community structure. The objectives of this study were to (1) compare the differences in diversity indices of understory plant, stand spatial structure and soil physiochemical properties between pure *P. massoniana* forests and different mixed *P. massoniana* afforested areas; (2) explore the response of different soil physiochemical properties and understory plant to stand spatial structure after different mixed afforestation; and (3) determine the factors affecting the differences in understory plant diversity after afforestation.

## 2 Materials and methods

### 2.1 Study area description

The experimental site is in Guang’an City, Sichuan Province, China (106°45′59″E-106°46′12″E,30°17′35″N-30°17′42″N, **Supplementary Figure S1**), which belongs to the parallel ridge and valley area of eastern Sichuan. This area is adjacent to the Jialing River, a primary tributary of the Yangtze River, and is an important ecological barrier in the upper reaches of the Yangtze River. Its climate can be classified as subtropical monsoon, with abundant rainfall, high air humidity, less sunshine, and a short frost period (Yin et al., 2022). The average annual temperature is 16°C, and ranges from 3°C in January to 33°C in July. The mean annual precipitation is 1200 mm with 43% of the total precipitation occurring in summer.

Three different types of stands were employed in the research area: pure *P. massoniana* forest (MPF), *P. massoniana*-*C. lanceolata* mixed forest (MCLMF), and *P. massoniana*-*L. formosana* mixed forest (MLMF), all of which are artificially afforested. The MPF was afforested in 1994 using seedling afforestation, with an initial density of 2000 plants per hectare. The MCLMF was established in 1998 by randomly selecting 10 areas, and planting *C. lanceolata* seedlings on the original *P. massoniana seedling* land. Specifically, positions were chosen where the growth of *P. massoniana* seedlings were inferior even death to cultivate *C. lanceolata* seedlings. The MLMF was established in 1998 by randomly selecting 6 areas. *L. formosana* seedlings were planted on the original *P. massoniana* seedling land, choosing the positions of inferior growth of *P. massoniana* seedlings. In these 16 mixed areas, the initial planting ratio of *P. massoniana* to both *C. lanceolata* and *L. formosana* was set at 6:4. In order to control the same initial stand density among different afforestation plots, we randomly selected three areas in the original *P. massoniana seedling* land for *in-situ* replanting of *Pinus massoniana* seedlings. We removed those dead or weak *P. massoniana* seedlings and replanted new healthy *P. massoniana* seedlings *in situ*. Ultimately, the seedling density was maintained at 2,000 plants per hectare across all 19 experimental plots. Since then, no human intervention has occurred, allowing the forest to grow naturally. In August 1998, an experiment using completely randomized design was conducted. Nine plots were selected, each possessing similar elevation (605–654 m), same soil type (Haplustepts) and similar slope (15^°^–17^°^) within the permanent plots (**Table 1**). We collected soil samples in each plot using a five-point sampling method in the same year, and brought them back to the laboratory to determine the soil water content, PH, total nitrogen, total phosphorus and total potassium in each plot. There was no significant difference between the plots.

**Table 1:** The basic information of sample plots at the beginning of afforestation.

Basic Indexes (1998)	(MPF) *pinus massoniana* pure forest	(MCLMF) Mixed forest of *Pinus massoniana* and *Cunninghamia lanceolata*	(MLMF) *Pinus massoniana* and *Liquidambar formosana* mixed forest
Elevation (m)	654.17 ± 1.41	605.66 ± 6.12	642.33 ± 4.98
Slope	15.33 ± 2.62	16.25 ± 2.94	17.33 ± 0.94
Slope position	middle	middle	middle
Soil water content	0.23	0.19	0.29
pH	6.14 ± 0.19	6.11 ± 0.14	6.13 ± 0.10
Soil total nitrogen (g kg^-1^)	1.31 ± 0.07	1.32 ± 0.02	1.30 ± 0.04
Soil total phosphorus (g kg^-1^)	0.30 ± 0.01	0.29 ± 0.01	0.31 ± 0.02
Soil total potassium (g kg^-1^)	12.55 ± 0.3	12.88 ± 0.21	12.99 ± 0.19

The three types of stands are MPF, pure *Pinus massoniana* forest; MCLMF, *Pinus massoniana*–*Cunninghamia lanceolata* mixed forest; MLMF, *Pinus massoniana*–*Liquidambar formosana* mixed forest. Data shown are the mean ± standard deviation (n = 3).

### 2.2 Experimental design and soil sampling

**Figure 1:**
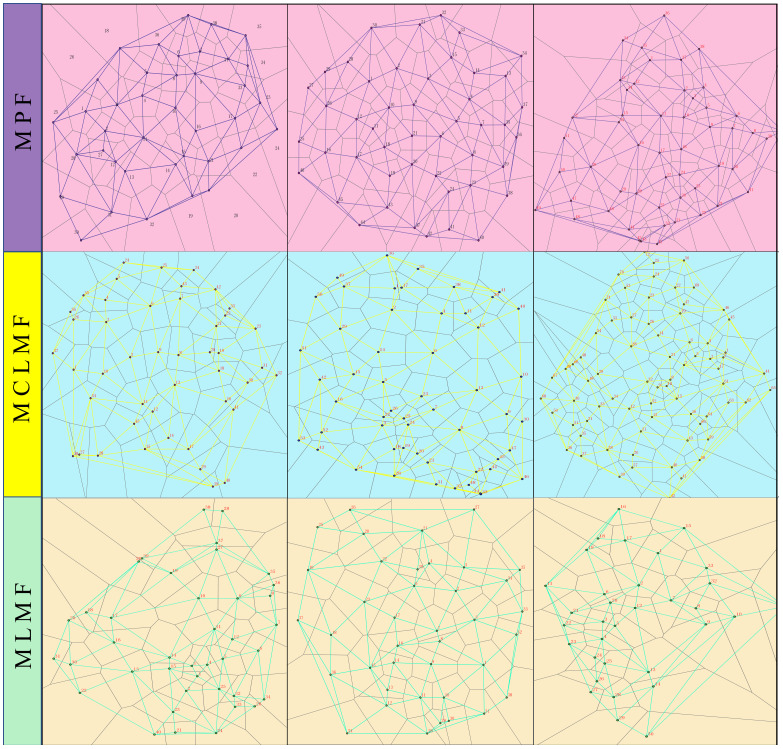
Visualization of Thiessen polygons of tree locations in different forest types. The three types of stands are MPF, mixed forest; MCLMF, mixed forest MLMF. In each forest type, there are three replicate plots, totaling nine plots. The Thiessen polygon network generated from the tree coordinates of the 9 plots, where the intersection point represents the tree at that location, and the polygon is the domain of each tree. The number and location of neighboring trees of each tree are obtained by the ARCGIS software through the interpretation of the Thiessen polygon network.

In August 2022, the measurement of a 20 m×20 m square plot was performed in each plot using a theodolite and a tape measure, with an additional 5-meter extension as the edge wood area. A total of nine square plots consisted of three pure *P. massoniana* forests (DBH, mean ± standard deviation: 19.63 ± 0.95 cm), three mixed forests of *P. massoniana* and *C. lanceolata* (DBH, mean ± standard deviation: 19.39 ± 1.13 cm), and three mixed forests (DBH, mean ± standard deviation: 24.02 ± 1.34 cm) of *P. massoniana* and *L.* formosana (**Supplementary Table S1**). PVC pipes were inserted at the four corners of each plot to serve as permanent markers. The GPS was employed to ascertain the coordinates of the central point (x_0_, y_0_) within the forest stand. Theodolite and measuring poles were used to determine the slope, azimuth, and distance of each tree in relation to (x_0_, y_0_). Subsequently, the software ArcGIS 10.7 was employed to determine the relative coordinates of each tree (x_1_, y_1_)… (x_n_, y_n_). The tree measuring ruler was used to measure the diameter at breast height, while the laser rangefinder and compass eyepiece elevation angle were used to determine the height of each tree (He et al., 2022). The creation of Thiessen polygons (**Figure 1**) involves conducting a neighborhood analysis to partition the adjacent trees surrounding each individual tree (Dong, 2008). Finally, the following formulas (**Table 2**) were used to calculate the stand spatial structure indexes:

**Table 2:** Equations for calculating the spatial structure characteristic indexes.

Spatial structure characteristics	Equation	Where	References
Mingling index	$M_{i}=\frac{1}{\text{n}}\sum_{\text{j}=1}^{\text{ n}}\text{Vij}$	$Vij=\{\frac{1}{0}$ , $\frac{\text{Species i}\neq \text{ Species j}}{\text{Otherwise}}$	(Hui et al., 2018)
Competition index	$CI_{i}=\sum_{j=1\;}^{\;\;n}\frac{Dj}{DiLij}$		(He et al., 2022)
Neighborhood comparison	$U_{i}=\frac{1}{n}\sum_{j=1}^{\;\;n}Kij$	$Kij=\frac{1}{0}$ , $\frac{Di<Dj}{Otherwise}$	(Gadow et al., 2012)
Uniform angle index	$W_{i}=\frac{1}{n}\sum_{j=1}^{\;\;n}Zij$	$\text{Zij}=\frac{1}{0}$ , $\frac{\text{αij}<\text{α}0}{Otherwise}$	(He et al., 2022)
Opening degree	$O_{i}=\frac{1}{n}\sum_{j=1\;}^{\;\;n}\frac{Lij}{Hij}$		(Hui et al., 2019)

Where, n is the number of neighbors, and in this study, The value of n comes from the Thiessen polygon; Di is the diameter at breast height (DBH) of the ith reference tree; Dj is the DBH of the jth neighboring tree; Lij is the distance between the ith reference tree and the jth neighboring tree; Hij is the height of the jth neighboring of tree i; αij is the horizontal angle between the reference tree and n neighboring trees; a standard angle α0 = 360°/n + 1. All the structure- based indices excepted *W*_i_ have five possible values intervals: 0, (0, 0.25], (0.25, 0.5], (0.5, 0.75], (0.75, 1]. The index *M*i in these five value intervals means zero, weak, moderate, strong and extremely strong mixed respectively. The *CI*_i_ in these five value intervals means zero, weak, moderate, strong and extremely strong compete respectively. The five value ranges of index *U*i respectively mean that the trees are in a dominant, sub-dominant, moderate, inferior and absolutely inferior state in the spatial structure unit. The *O*_i_ in these five value intervals respectively means five states of serious shortage, shortage, basic adequacy, adequacy and very adequacy of stand light transmission conditions. *W*_i_ is the angle scale of the ith reference tree, and its possible value ranges are [0, 0.475], (0.475, 0.517], and (0.517, 1], which respectively mean uniform distribution, random distribution, and clustered distribution.

Soil samples were collected at five randomly arranged S-shaped sampling lines in each plot after removing the surface debris of the soil, using a soil drill with a diameter of 5 cm. Since the soil layer in the experimental area is relatively thin (0–50 cm), the roots of understory plants are mainly distributed in the depth range of 0–30 cm from the soil profile dug on site, the soil sampling depth was set finally at 0–30 cm. The soil samples from the same plot were mixed evenly and packed into plastic bags and brought back to the laboratory for the determination of chemical properties. The sample for soil physical properties measurement was accomplished by a ring knife and aluminum box. A ring knife was used at the central point of the diagonal of each plot, as well as at a quarter of both left and right sides, to take out the *in-situ* soil and transferring it in to a portable refrigerator. In each plot, total of five ring knives were used in each plot, which were used to determine the soil bulk density (SBD) and porosity (SP). About 10g of the evenly mixed soil sample in the plastic bag was put into the pre-weighed and numbered aluminum box, the lid was covered and put into the refrigerator, and it was brought back to the laboratory within 24 hours.

### 2.3 Undergrowth species survey

The understory plant species survey was conducted in early September 2022. Five 5 m × 5 m quadrats were set up at the center and four corners of the 9 plots for the investigation of shrubs, recording the species, plant height, coverage, and crown width of each shrub. In the four triangular areas formed by the diagonals of the plot, 1 m×1 m quadrats were set up, with three in each triangular area, totaling 12, for the investigation of herbaceous plants.

### 2.4 Determination of physicochemical properties of soil samples

The physical properties of soil included soil temperature (ST), soil moisture (SM), soil bulk density (SBD), soil porosity (SP), and soil water content (SW). Soil temperature and moisture were measured directly at the soil sampling point in each plot, using a thermometer and hygrometer. The soil bulk density and porosity were determined by drying the soil ring knife which was sampled from the plot at 120°C and then weighed. The soil water content was determined using the “aluminum box weighing method”. The chemical properties measured in the laboratory included pH, total nitrogen (TN), available nitrogen (AN), total phosphorus (TP), available phosphorus (AP), total potassium (TK), available potassium (AK), and organic carbon content (SOC), which were referred to the previous reports (Xiang et al., 2024; Lyu et al., 2021; Yin et al., 2022).

### 2.5 Statistical analyses

The four indices, namely Shannon-Wiener index (H), richness index (R), Simpson index (D) also known as dominance index, and Pielou’s Index (J) also known as evenness index, were calculated using the package iNEXT in R 4.3 software to determine the diversity of understory plants (Hsieh et al., 2016). The calculation of important value (IV), niche width and niche overlap index, were referred to the previous reports (Wasof et al., 2015; Lyu et al., 2021). Before performing the differences analysis, the normality and variances homogeneity of data were tested by software EXCEL 2021 and SPSS V27.0. The Shapiro-Wilk test and Levene’s test were used for these tests. If the data did not meet normality, it was processed using z-score standardization. One-way ANOVA and multiple comparisons using Least Significant Difference (LSD) were used to determine differences among treatments in diversity, stand spatial structure indices, and soil physical and chemical properties. The F-test was used to perform significance tests on the difference results, and the significance result *p* value was set at< 0.05. The software ArcGIS 10.7 was used to perform neighborhood analysis on the point distribution of trees in each plot and to created Thiessen polygons (**Figure 1**). ‘Pearson correlation’, redundancy analysis (Lai et al., 2022), and ‘SEM’ (He et al., 2022) were used to analyze the relationship among stand spatial structure characteristic indexes (such as M, CI, U, W, and O), environmental factors (such as ST, SM, SOC, TN, AN, TP, AP, TK, AK, SW, PH, SBD, and SP) and plant diversity of shrubs and herbaceous (such as D, H, J, and R) in R 4.3 software (R Foundation for Statistical Computing, Vienna, AT) with packages “psych”, “pheatmap”, “rdacca.hp”, “tidyverse”, “lavaan”, “iNEXT”, and “ggcorrplot”. These data were 0–1 normalized and Collinearity diagnose was conducted in R 4.3 software with package “tidyverse” before RDA and SEM. To decrease collinearity, variables with the highest variance inflation factor (vif) values were removed one by one until the vifs of all variables in the RDA were lower than 10. Multiple tests were conducted to assess the model’s fitness after SEM (He et al., 2022; Lyu et al., 2022). Tests included chi-square tests, evaluation of the obtained p-value (χ2, *P* > 0.05 for a satisfactory fit), determination of the comparative fit index (CFI, CFI > 0.9 for a satisfactory fit), Root Mean Square Residual (SRMR, SRMR< 0.05 for a satisfactory fit), goodness-of-fit (GFI, GFI > 0.9 for a satisfactory fit), and root square mean error of approximation (RMSEA, RMSEA< 0.1 for a satisfactory fit).

## 3 Results

### 3.1 Changes in spatial structure characteristic indexes and environmental factors after mixed afforestation

**Figure 2:**
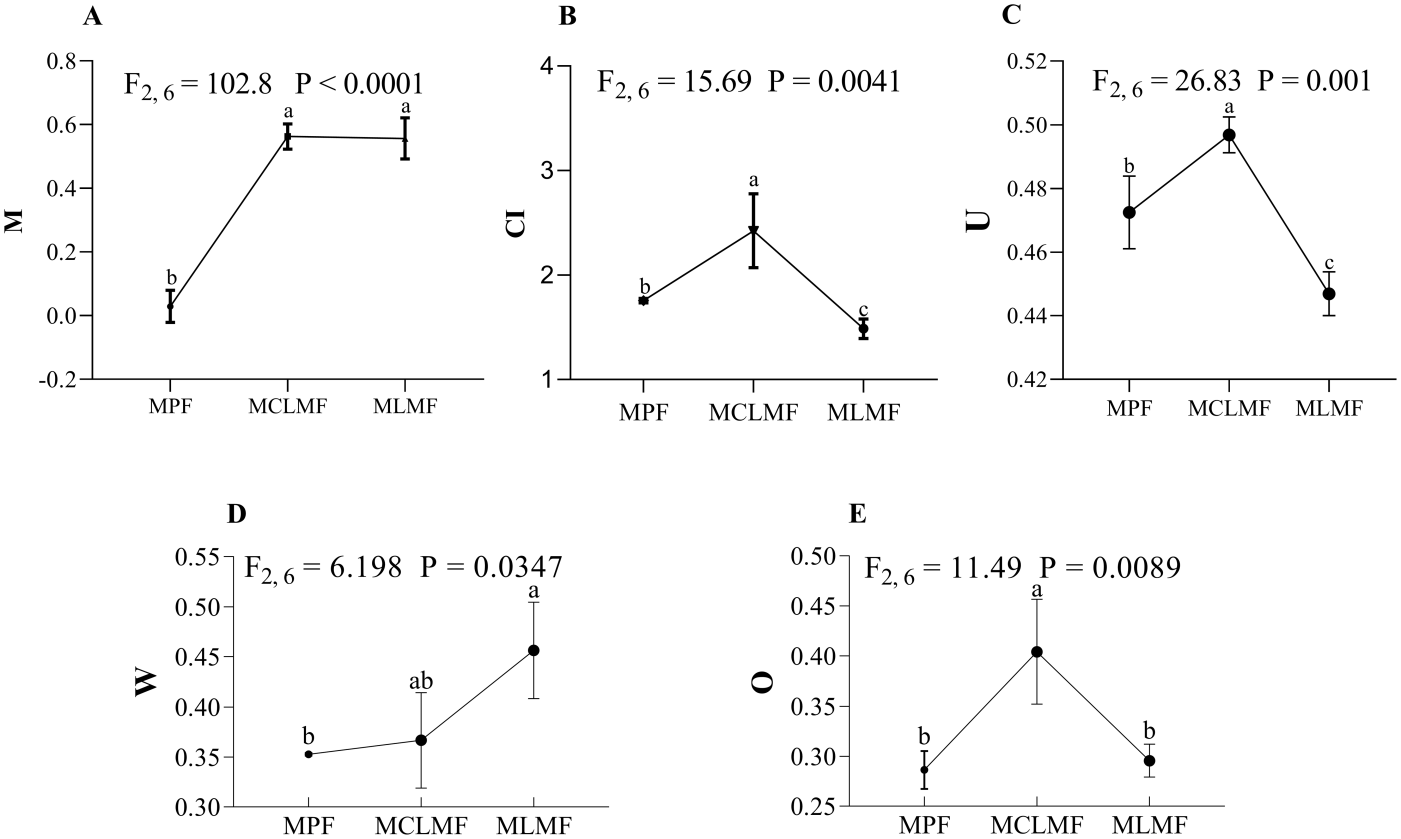
The stand spatial structure characteristic index. The three types of stands are MPF, pure *Pinus massoniana* forest; MCLMF, *Pinus massoniana*-*Cunninghamia lanceolata* mixed forest; MLMF, *Pinus massoniana*-*Liquidambar formosana* mixed forest. The stand spatial structure parameters are M, mingling index; CI, competition index; U, neighborhood comparison; W, uniform angle index; and O, opening degree. Data shown are the mean ± standard deviation (n = 3). These uppercase letters A-E represent the five subfigures in Figure 2. Different lowercase letters indicated that one of stand spatial structure indexes exerted a significant difference among the treatments of three types of stands (*P*< 0.05) in **(A–E)**.

There were significant differences in spatial structure characteristics among these three types of stands (**Figure 2**). Although there was no difference in the mingling index M between MCLMF and MLMF, they were both significantly higher than the pure stand (MPF). The competition index (CI) and neighborhood comparison (U) both showed mixed stand MCLMF > MPF > MLMF, indicating that MLMF mixed afforestation has weakened the competition among neighboring trees within the stand. Both the uniform angle index (W) and opening degree index (O) of the stand had significantly difference among MPF, MCLMF and MLMF, and they among different afforestation types ranked as MLMF > MCLMF > MPF and MCLMF > MLMF > MPF, respectively.

**Figure 3:**
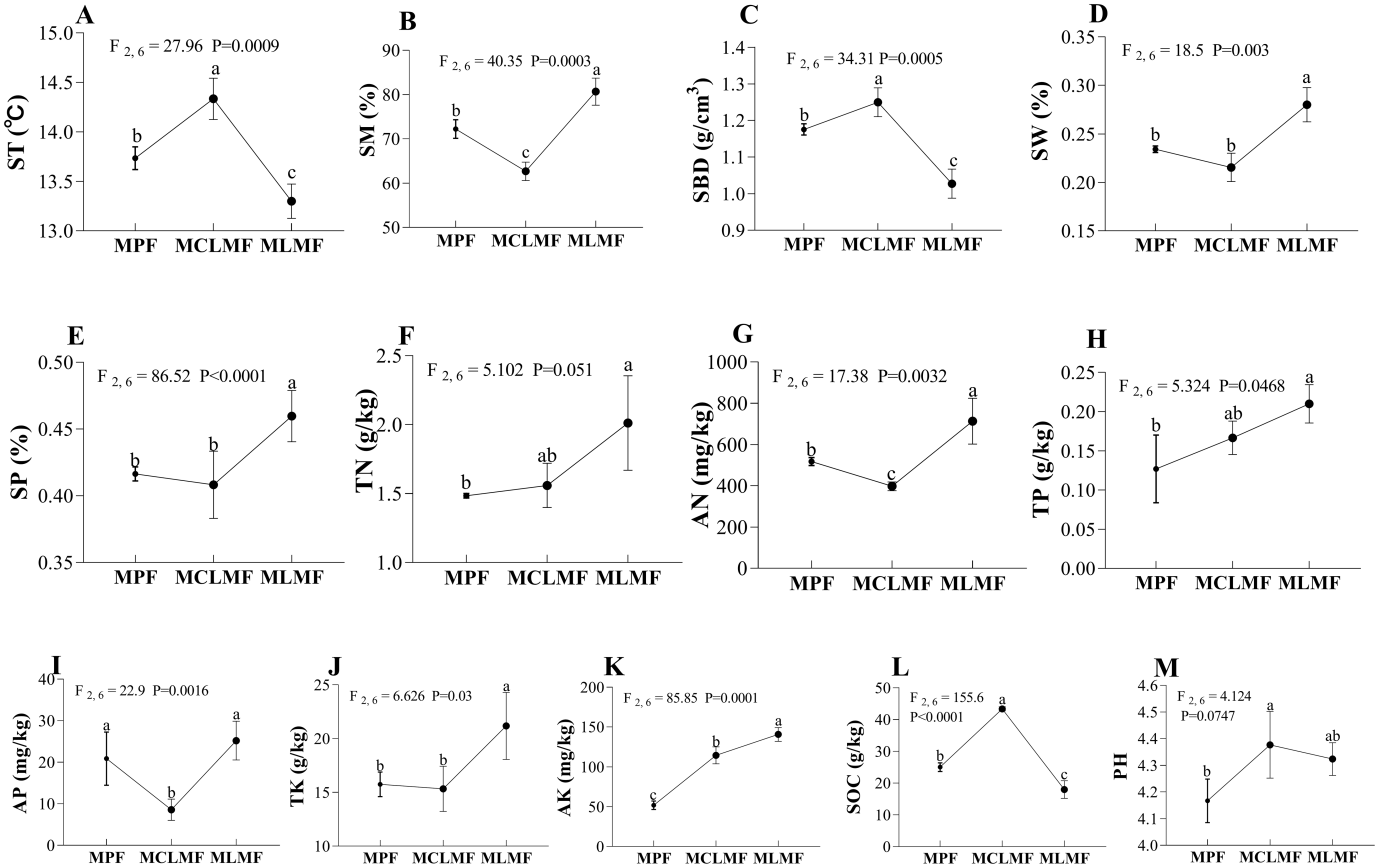
The environment factors under the three different afforestation types. The three types of stands are MPF, pure *Pinus massoniana* forest; MCLMF, *Pinus massoniana*-*Cunninghamia lanceolata* mixed forest; MLMF, *Pinus massoniana*-*Liquidambar formosana* mixed forest. The soil parameters are ST, soil temperature; SM, soil moisture; SBD, soil bulk density; SW, soil water content; SP, soil porosity; TN, soil total nitrogen; AN, soil available nitrogen; TP, soil total phosphorus; AP, soil available phosphorus; TK, soil total potassium; AK, soil available potassium; SOC, soil organic carbon; pH, soil pH. Data shown are the mean ± standard deviation (n = 3). Different lowercase letters indicated significant differences among the treatments (*P*< 0.05) in **(A–M)**.

After mixed afforestation, significant changes occurred in the physicochemical properties of the stand soil. Except for TN, TP, and pH, other physicochemical properties [e.g., ST (F_2,6_ = 27.96, *P*< 0.01), SM (F_2,6_ = 40.35, *P*< 0.01), SBD (F_2,6_ = 34.31, *P*< 0.01), SW (F_2,6_ = 18.5, *P*< 0.01), AN (F_2,6_ = 17.38, *P*< 0.01), AP (F_2,6_ = 22.9, *P*< 0.01), AK (F_2,6_ = 85.85, *P*< 0.01), SOC (F_2,6_ = 155.6, *P*< 0.01)] showed significant differences among the three types of stands (*P*< 0.05, **Figure 3**). MCLMF increased ST, SBD, AK, SOC, and pH, and decreased AN and AP. MLMF increased SH, SW, SP, AN, TK, and AK, and decreased ST, SBD, and SOC.

**Figure 4:**
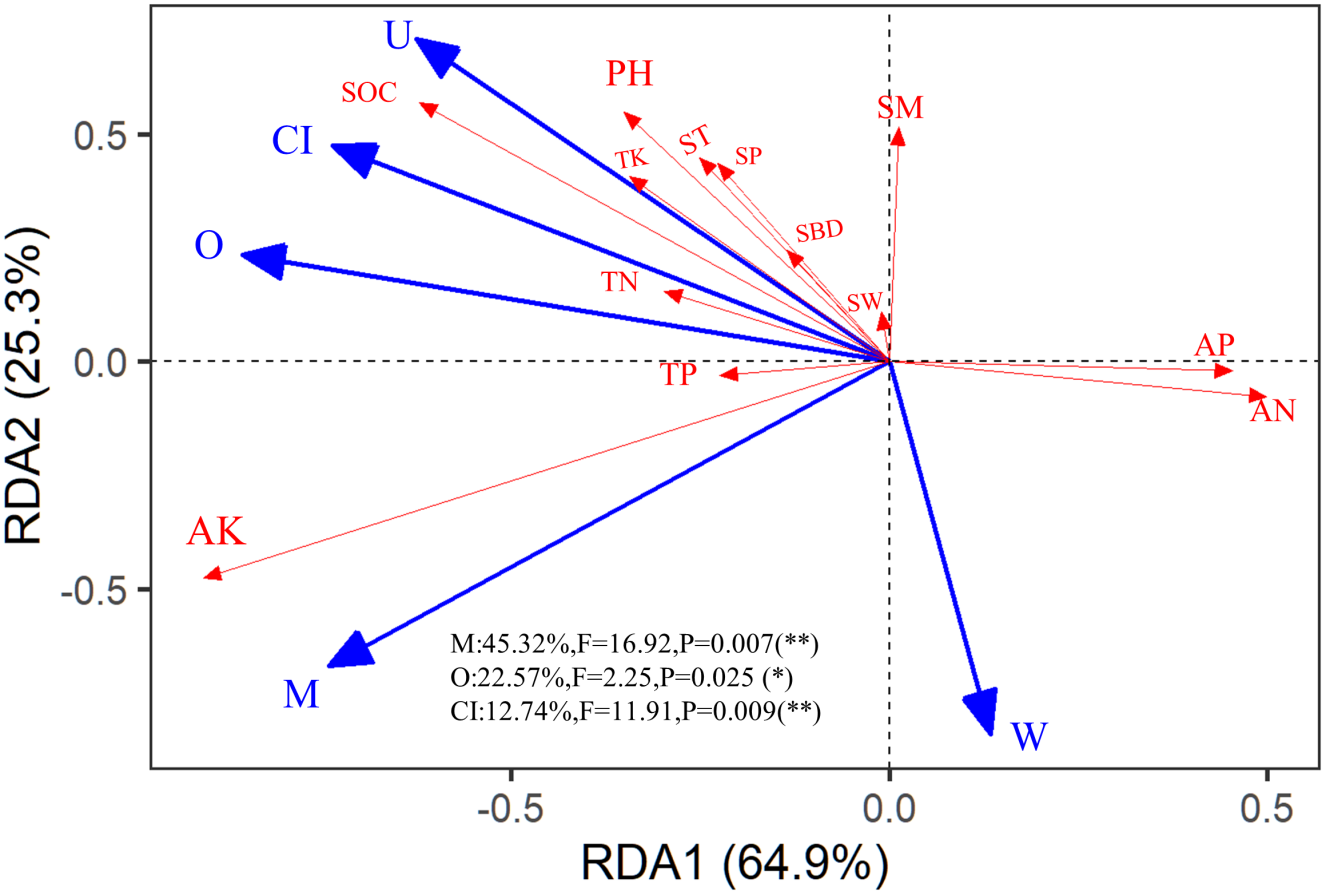
Redundancy analysis (RDA) among the stand spatial structure characteristic indexes and environmental factors. The angle between the two indicators represents a significant and positive relationship (acute angle, < 90°) or negative relationship (obtuse angle, > 90°). The dot product of two-line segment vectors is proportional to the standardized path coefficient. The stand spatial structure parameters are M, mingling index; CI, competition index; U, neighborhood comparison; W, uniform angle index; and O, opening degree. The soil parameters are ST, soil temperature; SM, soil moisture; SBD, soil bulk density; SW, soil water content; SP, soil porosity; TN, soil total nitrogen; AN, soil available nitrogen; TP, soil total phosphorus; AP, soil available phosphorus; TK, soil total potassium; AK, soil available potassium; SOC, soil organic carbon; pH, soil pH.

Since all spatial indices had significant correlations with environmental factors as shown in **Supplementary Figure S2**, a redundancy analysis (RDA) was conducted on the five spatial structure indices and 13 physicochemical properties. The explanations (exp) for the first and second axes of the RDA were 64.90% and 25.30%, respectively (**Figure 4**). M (con = 45.32%, F = 16.92, *P* = 0.007); O (con = 22.57%, F = 2.25, *P* = 0.03), and CI (con = 12.74%, F = 11.91, *P* = 0.009) had significant contributions to the changes in soil physicochemical properties.

### 3.2 Changes in the structure of the shrub and herb community under the stand

**Figure 5:**
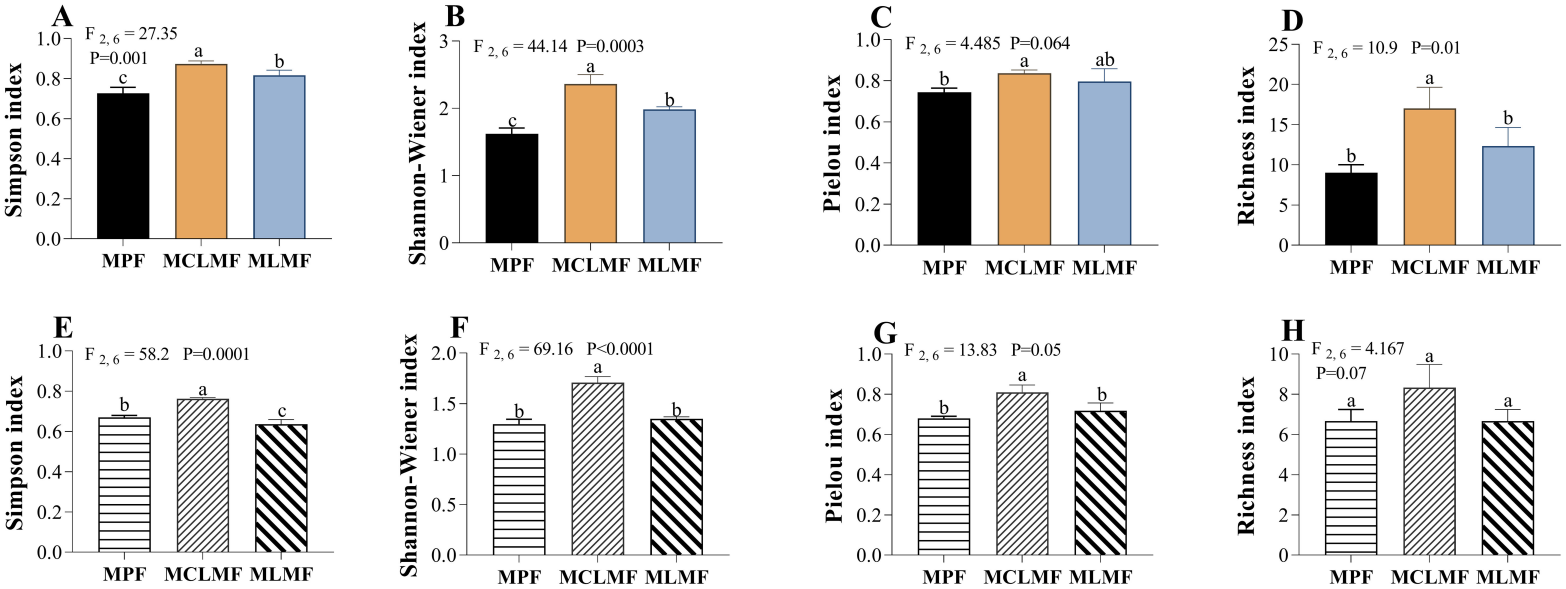
The diversity of understory shrub communities and herbaceous communities under different afforestation types. **(A–D)** represent the diversity of shrub communities, while **(E–H)** represent the diversity of herbaceous communities. The three types of stands are MPF, pure *Pinus massoniana* forest; MCLMF, *Pinus massoniana*–*Cunninghamia lanceolata* mixed forest; MLMF, *Pinus massoniana*–*Liquidambar formosana* mixed forest. The diversity indexes are simpson index (dominance index), shannon-wiener index (diversity index), pielou index (eveness index) and richness index. Data shown are the mean ± standard deviation (n = 3). Different lowercase letters indicate significant differences among the treatments (*P*< 0.05) in **(A–H)**.

The diversity indices of shrubs showed in (**Figures 5A–D**). The Simpson index (F_2, 6_ = 27.35, *P*< 0.01) and Shannon-Wiener index (F_2, 6_ = 44.14, *P*< 0.01) of the community had significant differences among the three types of stands (*P*< 0.05), and the order of their index’s values among the three afforestation types was MCLMF>MLMF>MPF. There was a significant difference between MCLMF and MPF, regarding the pielou index (evenness) of the community, but there was no difference between the other two (between MPF with either MCLMF or MLMF). The shrub community richness index showed that the MCLMF was significantly larger than the other two types of stands. There was a significant difference in the Simpson index (F_2,6_ = 58.20, *P*< 0.01) of the herb communities among the three types of stands (**Figures 5E**; *P*< 0.05), and the value of Simpson order among the three afforestation types was MCLMF > MPF > MLMF. The Shannon-Wiener index (F_2,6_ = 69.16, *P*< 0.01) and evenness index (F_2,6_ = 13.83, *P* = 0.05) of the community both showed that the herb diversity of MCLMF was significantly larger than that of the other two types of stands (**Figures 5F, G**; *P*< 0.05). At the same time, there was no difference between MPF and MLMF. There was no significant difference among the three types of stands considering the richness index.

As shown in **Supplementary Table S2**, mixed afforestation significantly impacted the importance value and ecological niche structure of the shrub community under the stand. In MPF, the dominant species were *Rubus buergeri* and *Eurya japonica*, generalist species with a broad ecological niche. In MCLMF, the dominant species are *R. buergeri* and *Smilax china*. In MLMF, the dominant species were *R. buergeri*, *Maesa japonica*, and *Millettia oosperma*. Mixed afforestation impacted the importance value and ecological niche structure of the herb community under the stand, as shown in **Supplementary Table S3**. In MPF, *Dicranopteris pedata*, *Pteridium aquilinum*, and *Miscanthus sinensis* were the dominant species. In MCLMF, *D. pedata* and *P. aquilinum* were the dominant species in the community. *Iris japonica* was the dominant species in the MLMF herb community, with its importance value accounting for 42% of the entire community.

**Figure 6:**
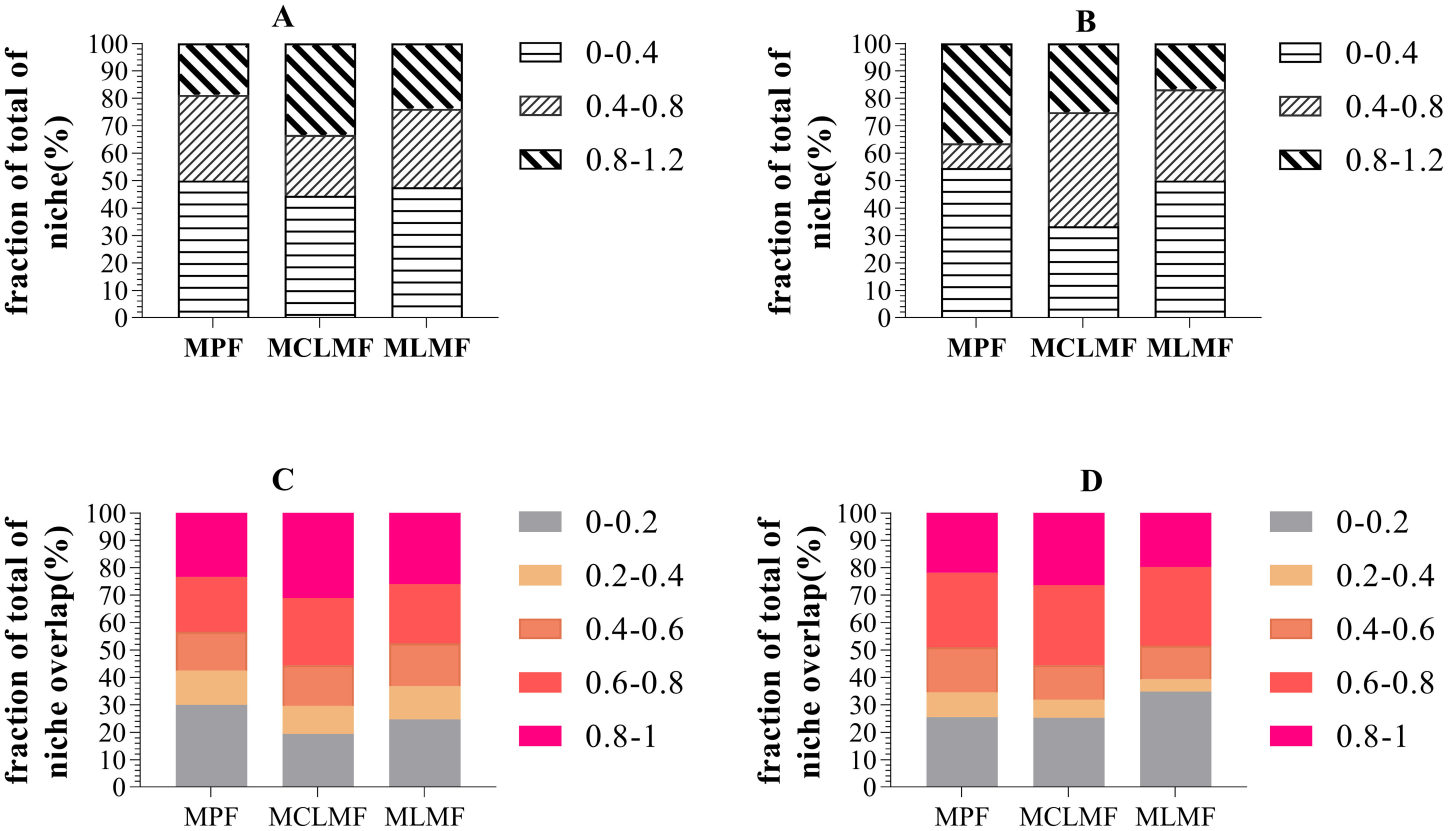
The relative abundance of the niche width and resource overlap of understory shrubs and herbs. **(A, B)** represent the relative niche width of shrubs and herbs communities respectively, while **(C, D)** represent the relative resource overlap of shrubs and herbs communities respectively. In order to have a more intuitive understanding of the distribution of the ecological niche width index set and the ecological niche overlap index set of understory plant communities in the three forest classification types. We divided the ecological niche width values of various plants in three forest stand types into three numerical intervals in proportion from the lowest to the highest: [0, 0.4), [0.4, 0.8), [0.8, 1.2]. We also divided the ecological niche overlap index values into five numerical intervals in proportion from the lowest to the highest: [0, 0.2), [0.2, 0.4), [0.4, 0.6), [0.6, 0.8), [0.8, 1.0]. The three types of stands are MPF, pure *Pinus massoniana* forest; MCLMF, *Pinus massoniana–Cunninghamia lanceolata* mixed forest; MLMF, *Pinus massoniana–Liquidambar formosana* mixed forest.

**Figure 6** shows the proportions of niches at all levels, with both MCLMF and MLMF having a more significant proportion of high niche width than MPF in terms of shrub communities and a higher overlap of species resources than MPF. Regarding herbaceous communities, compared with MPF, MCLMF and MLMF increased their proportion of medium niche width. MCLMF increased the proportion of resources high-overlap species, while MLMF increased the proportion of low-overlap species.

### 3.3 The relationships among understory plant diversity, spatial structure characteristic indexes, and environmental factors

The results showed that D1 (Simpson index of shrubs) was significantly and positively correlated with M, O, pH, and AK but significantly and negatively correlated with AP (*P*< 0.05, **Supplementary Figure S3**). H1 (Shannon-wiener index of shrubs) was significantly and positively correlated with M, O, PH, and SOC but significantly and negatively correlated with AP (*P*< 0.05, **Supplementary Figure S3**). J1 (Pielou’s index of shrubs) was significantly and positively correlated with M (*P*< 0.05, **Supplementary Figure S3**). R1 (richness index of shrubs) was significantly and positively correlated with M, O, pH, SOC, and ST but significantly and negatively correlated with AP and SM (*P*< 0.05; **Supplementary Figure S3**). D2 was significantly and positively correlated with CI, O, SOC, ST, and SBD but significantly and negatively correlated with SM, AP, SW, AN, and SP (*P*< 0.05; **Supplementary Figure S3**). H2 was significantly and positively correlated with CI, O, U, SOC, and ST but significantly and negatively correlated with SM and AP (*P*< 0.05; **Supplementary Figure S3**). J2 was significantly and positively correlated with CI, O, SOC, and ST (*P*< 0.05; **Supplementary Figure S3**). R2 was significantly and positively correlated with O and SOC but significantly and negatively correlated with SM, AP, and SW (*P*< 0.05, **Supplementary Figure S3**).

**Figure 7:**
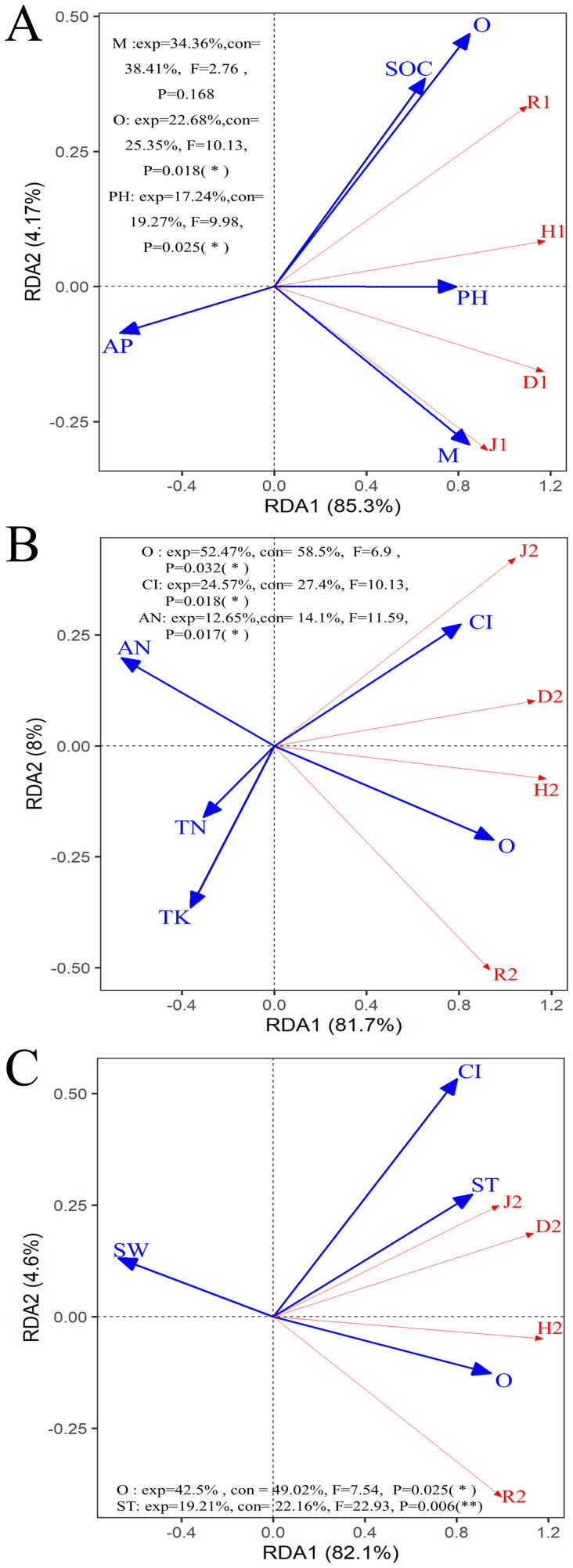
Redundancy analysis (RDA) among the stand spatial structure characteristic indexes, environmental factors, and biomass allocation of organs. **(A)** represents the diversity of shrub communities, while **(B, C)** represent the diversity of herbaceous communities. The angle between the two indicators represents a significant and positive relationship (acute angle, < 90°) or negative relationship (obtuse angle, > 90°). The dot product of two-line segment vectors is proportional to the standardized path coefficient. The stand spatial structure parameters are M, mingling index; CI, competition index; and O, opening degree. The soil parameters are ST, soil temperature; SW, soil water content; TN, soil total nitrogen; AN, soil available nitrogen; AP, soil available phosphorus; TK, soil total potassium; AK, soil available potassium; SOC, soil organic carbon; pH, soil pH. The diversity parameters are: D1–2, Simpson index; H1–2, Shannon-wiener index; J1–2, pileous index and R1–2, richness index, number 1 and 2 represent shrub communities and herbaceous communities, respectively.

The overall explanation rate was 89.47% (Axis 1 = 85.3%, Axis 2 = 4.17%) in the RDA results for the shrub community (**Figure 7A**). Among them, M was the most significant contribution, accounting for 38.41% of the model explanation rate and explaining 34.36% of the changes in the shrub community. The second most important contribution was from O, with contribution and explanation rates of 25.35% and 22.68%, respectively. This result indicates that the more changes in openness of the stand space, the more changes in light and temperature would cause changes in the original types and quantities of understory shrubs. The third significant contribution was soil pH, with contribution and explanation rates of 19.27% and 17.24%, respectively.

As shown in **Figure 7B**, the ranking of the significant contributions of the selected spatial structure indices and soil chemical properties to the herb community was O (exp = 52.47%, con = 58.5%, F = 6.90, *P* = 0.032), CI (exp = 24.57%, con = 27.9%, F = 10.13, *P* = 0.018), and AN (exp = 12.65%, F = 11.59, con = 14.1%, *P* = 0.02). These three variables explained 89.69% of the changes in the herb community structure. As shown in **Figure 7C**, the ranking of the significant contributions of the selected spatial structure indices and soil physical properties to the herb community was O (exp = 42.50%, F = 7.54, con = 49.02%, *P* = 0.03) and ST (exp = 19.21%, con = 22.16%, F = 22.93, *P*< 0.01). Together, these two variables explained 61.71% of the changes in the herb community structure.

**Figure 8:**
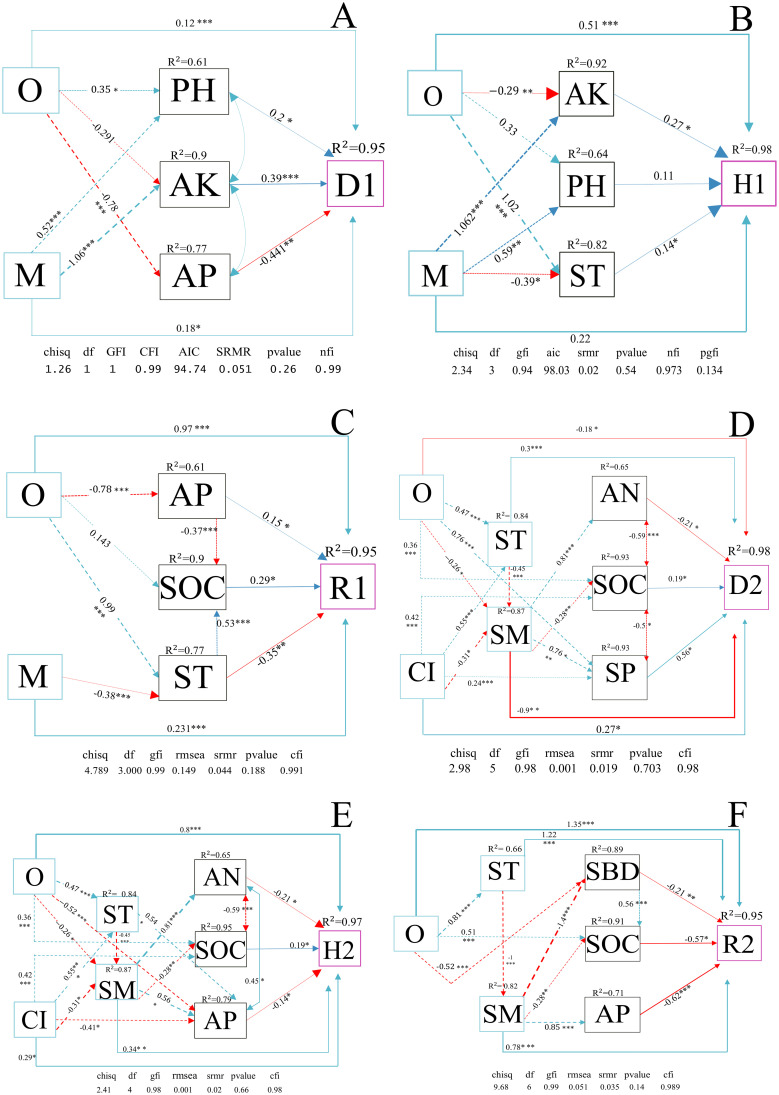
**(A–F)** Structural equation models (SEMs) of the relationships between the diversity indices of understory shrubs and herbs, forest spatial structure characteristic indices, and environmental factors. **(A–C)** represent the diversity indices of shrubs, while **(D–F)** represent the diversity indices of herbs. The solid and dashed arrow are the direct and indirect pathways, respectively. The blue and red arrow represent significant positive and negative effects, respectively. The thickness of the arrows is proportional to the magnitude of the standardized path coefficients. R^2^ values associated with response variables indicate the proportion of explained variation by relationships with other variables. ***P< 0.001; **P< 0.01; *P< 0.05. The diversity parameters are D1–2, Simpson index; H1–2, Shannon-wiener index; R1–2, richness index, number 1 and 2 represent shrub communities and herbaceous communities, respectively. The stand spatial structure parameters are M, mingling index; CI, competition index; U, neighborhood comparison; W, uniform angle index; and O, opening degree. The soil parameters are SOC, soil organic carbon; AN, soil alkaline nitrogen; AP, soil available phosphorus; AK, soil available potassium; ST, soil temperature; SM, soil moisture; pH, soil pH; SBD, soil bulk density; SP, soil porosity.

As shown in **Figures 8A–C**, three figures were structural equation models (SEM) of the direct and indirect effects of the stand spatial structure factors and soil physicochemical property factors identified in the previous text on the diversity of shrubs. Obviously, O indirectly affected D1 and H1 through pH (D1: 0.2, *P*< 0.05; H1: 0.11, *P* > 0.05) and AK (D1: 0.39, *P*< 0.001; H1: 0.27, *P<* 0.05). M indirectly affected D1 and H1 through pH (0.20, *P*< 0.05) and AK (D1: 0.39, *P*< 0.01; H1: 0.27, *P*< 0.05). O indirectly affected R1 and D1 through AP (R1: 0.15, *P*< 0.05; D1: −0.441, *P*< 0.01). M indirectly affected R1 and H1 through ST (R1: −0.35, *P*< 0.01; H1: 0.14, *P*< 0.05) and SOC.

As shown in **Figures 8D–F**, these three figures were SEM of the direct and indirect effects of the stand spatial structure factors and soil physicochemical property factors screened previously on the diversity of herbs. O had a direct negative impact on D2 (−0.177, *P*< 0.05) and SM (−0.26, *P*< 0.05), a direct positive effect on H2 (0.8, *P*< 0.01), R2 (1.35, *P*< 0.01), ST (0.47, *P*< 0.001), and SOC (0.36, *P*< 0.01). CI has a direct positive effect on D2 (0.27, *P*< 0.05), ST (0.55, *P*< 0.01), H2 (0.29, *P*< 0.05), and SOC (0.42, *P*< 0.01) and a direct negative effect on SM (−0.31, *P*< 0.05) and AP (−0.41, *P*< 0.05). O produced an indirect impact on D2 through ST (0.3, *P*< 0.001), SP (0.56, *P*< 0.05), SM (−0.9, *P*< 0.01), and SOC (0.19, *P*< 0.05), an indirect effect on H2 through SOC (0.19, *P*< 0.05), SM (0.34, *P*< 0.01), and AP (-0.14, *P*< 0.05), an indirect effect on R2 through ST (1.22, *P*< 0.001), SOC (−0.57, *P*< 0.05), and SBD (−0.21, *P*< 0.01).

## 4 Discussion

### 4.1 Effects of mixed afforestation on stand spatial structure

The stand spatial structure indicate the actual situation of the tree layer in terms of the degree of mixed planting, competition status, distribution pattern, and spatial openness (Hui et al., 2018, 2019). The research in this article found that nearly 30 years after mixed planting, the mingling index in mixed forests had significantly increased, consistent with previous research conclusions (Wang et al., 2023), indicating that mixed afforestation did increase the species composition in the forest. The competition of trees in MCLMF intensified compared with MPF, while the competition of trees in MLMF weakened. This may be caused by differences in complementarity or overlap of interspecific ecological niches under these three different afforestation types (Wang et al., 2023). The ecological niches of the conifers *P. massoniana* and *C. lanceolata* were similar, increasing their competition. The final result was that *P. massoniana*, which had the first-mover advantage, achieved more robust and taller growth (Venail et al., 2014), intensified the subsequent expansion of resource competition between *P. massoniana* and *C. lanceolata*.

The MLMF is a mixed forest of *P. massoniana* and *Liquidambar formosana*, the two tree species have complementary ecological niches. The sizable light-receiving area of *L. formosana* leaves, which allows for specific advantages in competing for light and heat resources (Yao et al., 2015), somewhat offsets the early advantages of *P. massoniana*. Consequently, the different species in the mixed forest of *P. massoniana* and *L. formosana* complement the resources of each other, decreasing their competition with no noticeable growth differences occurring. The uniform angle index was used to describe the distribution pattern of the trees in the horizontal space. We observed that the horizontal distribution pattern of trees in MLMF was a random distribution, while the distribution of trees in MPF and MCLMF is a uniform distribution. Judging from the trend of numerical changes of index W, mixed afforestation does cause the change trend of distribution pattern of trees from uniform to random (**Figure 2**). This result has been confirmed in several previous studies (Hui et al., 2018; Dong et al., 2022). The O was the relative ratio of the openness of the stand spatial structure in the horizontal direction and the height of the forest layer in the vertical direction. Consequently, the greater the openness, the more sunlight can enter the understory (He et al., 2022). We also found that the openness in MCLMF was significantly larger than that of MPF and MLMF. However, there was no difference in the openness between MLMF and MPF. This result showed that MCLMF had more understory light than MPF and MLMF. The reason for this might be that the competition among tree species in the MCLMF is greater, leading to differentiation in tree growth, resulting in a larger height difference (Wang et al., 2023).

### 4.2 Effects of mixed afforestation on soil properties

Mixed planting causes the differentiation of stand spatial structures because trees affect the resource acquisition of each other through the selective development of organs during their growth process (Sevillano et al., 2016; Bebre et al., 2021). The particular development of organs in these trees allocates the space of the stand, thereby affecting the light, temperature, water, and heat of the understory (Luan et al., 2012). We found that the ST and SBD in MCLMF were significantly higher than those in MPF, but not in MLMF. The reason could be that the openness of MCLMF was significant, and more sunlight reached the ground, increasing ST and SBD accordingly due to its positive correlation with ST (Zhou et al., 2022). Previous studies have confirmed this relationship (Van Couwenberghe et al., 2011; He et al., 2022). SM and soil porosity (SP) in MLMF were significantly higher than both in MPF and MCLMF. This result may be because the competition between trees in MLMF was minimal. Specifically, the trees height of *P. massoniana* and *L. formosana* in MLMF were similar and the trees were tall, which resulted from Complementary niche and less competition between the two tree species (Wang et al., 2023). Therefore, the openness of MLMF was low, the entrance of sunlight into the understory was difficult. Soil moisture evaporation was minimal and occupying a specific soil space, and soil porosity was also significant (Nardini et al., 2021).

The sources of soil chemicals included weathering of parent material (Slessarev et al., 2019), atmospheric deposition (Chen et al., 2013), rainwater deposition, microbial activity (Whitaker et al., 2014), and plant litter (Hoyos-Santillan et al., 2018). These factors directly or indirectly affect the chemical properties of soil. The stand spatial structure affects the process of rainwater entering the understory because the canopy intercepts the flow (Raz-Yaseef et al., 2010). Additionally, the spatial structure of the stand affected the microbial activity and weathering of the parent material (Horak et al., 2014). We found that soil TN and TP significantly increased in MLMF compared to MPF. In contrast, MCLMF showed a slight increase. After analyzing our RDA results, we observed the growth effect of openness (O) and the Mingling index (M). This result indicates that mixed planting of MLMF could indeed increase the TN and phosphorus contents in the soil, in agreement with previous research (Gong et al., 2021a; Chen et al., 2020).

Soil AN and soil-available phosphorus (AP) showed an interesting phenomenon: compared with the MPF, soil AN and AP significantly increased in MLMF but decreased in MCLMF. This result may be from two reasons, Firstly, the light under MCLMF is sufficient resulted from higher openness (O), the understory plants grow vigorously, and understory plants take away a lot of available nutrients (Gutiérrez-Girón et al., 2014). In contrast, the light under the MLMF forest is shaded by the canopy due to the low stand spatial openness, the growth of understory plants is limited, and the available nutrients of the soil are not taken away in large quantities, so more available nutrients are retained. Secondly, it may be caused by the local subtropical monsoon climate with heavy summer rainfall (Yang et al., 2010). The understory soil in MCLMF with a more considerable O has a more obvious rainfall impact and leaching. Consequently, AN and AP, water-soluble substances in soil, are easily eroded and leached by rainfall (Cavagnaro et al., 2015). In contrast, MLMF plants have lush broad leaves in summer, blocking the impact and leaching of rainwater (Schumacher and Christiansen, 2015).

Soil organic matter is a manifestation of the forest carbon sink function in the underground part, and its content depends on how much productivity is transferred to the underground during the growth, development, and death of aboveground plants. The efficiency of this process is affected by light, temperature, water, heat, and soil biological activity (Zhang and Zhou, 2018; Sun et al., 2021).We found that the soil organic matter content of the three types of stands differed significantly. MCLMF had the most soil organic matter, followed by MPF and MLMF. MCLMF has significant openness in the tree layer. More sunlight reaches the understory, coupled with simultaneous rain and heat in summer, promoting the photosynthetic production of understory shrubs and herbs and more shrub and grass residues returning to the soil each year (Chen et al., 2020; Yang et al., 2022). The soil organic matter of MLMF decreased, related to less understory light (small openness), less competition in the tree layer, and fewer photosynthetic products of understory plants (Adie and Lawes, 2009). In summary, mixed planting significantly impacts the physical and chemical properties of understory soil. These impacts were driven by the standard spatial structure (e.g., M, O, and CI).

### 4.3 Effects of mixed afforestation on understory shrubs and herbs

After mixed management, we found that the shrub Shannon (H1) and Simpson (D1) indexes of MCLMF and MLMF were significantly higher than those of pure forest MPF. The richness R1 and evenness J1 indexes observed in MCLMF were significantly higher than in MPF and MLMF. This shows that after mixed planting, MCLMF improves the diversity of understory shrubs by increasing the number of species while ensuring balance and evenness among species without the appearance of prominent dominant species or differences in species number.

MLMF improved the diversity of understory shrubs by reducing the proportion of dominant species. However, MLMF did not achieve evenness among species, and an uneven number of species still existed. The stand spatial structures that significantly impacted shrub diversity were M and O (**Figure 7A**). This result shows that mixed management and increased understory light can enhance the variety of shrubs, consistent with previous research (Butler et al., 2008; Radhamoni et al., 2023). A more detailed inference from the above results was that the increase in O increased understory light, which enhanced the types and evenness of understory shrubs. At the same time, the mixed planting weakened the status of the dominant species in the shrub community.

We found that the richness of understory herbs was not affected in MLMF. Although the number of herb species in MCLMF has increased, it was insignificant (**Figures 5H, P** > 0.05). This may be because the shrub layer also affects the survival of herb community species and the influence of space size and light intensity (Prévosto et al., 2016). The shrub layer limits the expansion of the ecological niche of the herb layer, resulting in insignificant changes in the species richness of the herb layer (Toth, 2021). We found that the Shannon index and Simpson and evenness indexes of MCLMF were all significantly higher than those of MPF (**Figures 5E–G**, *P*< 0.05) This result shows that MCLMF improves the diversity of the herb community by enhancing the uniformity of the herb community and reducing the proportion of dominant species in the community.

O and CI were the main spatial features driving the structure of the herb community (**Figures 7B, C**). The reason may be that O improves the light environment of the understory of MCLMF, promotes the growth of herbs, and reduces the light restrictions to make the herb community more uniform. Still, the existence of shrubs as competitors limits the expansion of herb species. Consequently, species richness did not exert significant difference with that of MPF (Toth, 2021). There was no fierce survival competition in the tree layer (smaller CI index) in MLMF. Therefore, the trees grew strong and were tall, the light entering the understory was reduced and was first intercepted by shrubs. Although causes the light and heat conditions of the herb layer to decrease, however, the distance between trees increased, leaving more living space for herbs. Consequently, in the case of resource scarcity but ample living space, the herb community is fiercely competitive, eventually the dominant herb species *I. japonica* appeared in MLMF (**Supplementary Table S3**), which was consist with the result of **Figure 5E** that Simpson index in MLMF is lower than that in MPF. In summary, mixed planting significantly impacts the community structure of understory shrubs and herbs. These impacts were driven by the stand spatial structure (mainly O, M and CI).

### 4.4 Relationship among stand spatial structure, soil physicochemical properties, and the diversity of understory shrubs and herbs

We tested the direct or indirect effects of stand spatial structure characteristic indexes and environmental factors on the plant diversity of shrubs and herbs through SEM analysis. We found that soil chemical properties (e.g., pH, SOC, AK, and AP) were significantly correlated with shrub diversity. Except for AP, the remaining physicochemical properties promoted shrub diversity. This result was because the soil phosphorus content of *P. massoniana* plantations in southern China was generally insufficient. Consequently, stand with better soil P preservation can provide more favorable growth for the tree layer (Wang et al., 2022a). However, this may promote canopy coverage but decrease O and understory light, inhibiting the diversity of understory shrubs (Boothroyd-Roberts et al., 2013). This was also observed in the SEM analysis (**Figure 8**), in which O exerted a negative correlation with AP while a positive correlation with shrub diversity. The AK in the soil is easily absorbed and utilized during plant growth, and it is significantly positively correlated with the diversity and richness indexes of shrubs (Lyu et al., 2021). We found that M had a positive effect on shrub diversity and AK. The reason may be that increased plant diversity after mixed afforestation is often accompanied by a greater complexity of root system structure with various microbial function, which may indirectly promote the cycling and availability of trace elements such as potassium (Wang et al., 2021). *P. massoniana* has a foraging preference for soil NH^+^, it may lead to soil acidification that is detrimental to the growth of understory shrubs (He et al., 2022), and mixed planting can weaken the acidification effect of *P. massoniana* on soil. This was also observed in the SEM analysis, in which M indirectly promoted the Shannon index of shrubs through increased soil pH. Soil organic matter can improve soil fertility, promote microbial activity, accelerate the soil material cycle, produce a large amount of available nutrients that are beneficial to plant absorption (Satdichanh et al., 2023). We found that Soil organic matter had an increasing effect on the Shannon index and richness index of shrubs, which was regulated by O. The reason may be that stand openness affected the understory hydrothermal environment, accelerating the rate of nutrient cycling (Henneron et al., 2020). In summary, M and O, as well as soil pH, SOC, AK, and AP, were essential driving factors affecting the community structure of shrubs. These factors affected the species composition, quantity proportion, and spatial distribution of shrubs through various direct and indirect action paths.

We found that ST and SBD were positively correlated with herb diversity, and O and CI regulated this association. When the competition between trees in the tree layer was intense, the growth of trees was differentiated, the canopy was stratified, the openness of the space was improved, the understory light was enhanced, and the herbs thrived (Churski et al., 2022; Xiang et al., 2024). According to SEM, SBD had a direct negative correlation with herbs. Still, because it was indirectly negatively regulated by O, the combined effect of O and SBD enhanced herb diversity. We found that the competition index CI of the tree layer had a direct positive effect on the diversity of the herb community. This result is consistent with previous research results (Li et al., 2014). One study indicated that logging could promote the growth of understory seedlings by reducing the competition of neighboring trees (He et al., 2022). However, we found that the competition of neighboring trees directly promoted understory herbs. There are two reasons for this difference. The first reason was that our research objectives were different. They studied the growth of seedlings, while we explored the diversity of herbs (He et al., 2022). The second explanation was that the reasons for environmental improvement were different. They increased the survival space of understory seedlings by logging, while the MCLMF in our research improved understory plants light condition by causing the canopy of trees to stratify due to competition through a naturally formed spatial structure (Churski et al., 2022). There was a fundamental phenomenon in the indirect effect of stand space on herbs: both O and CI promoted the diversity of herbs through SOC (**Figure 8**). This result may have occurred given the long-term environmental shaping (O and CI), the synergistic increase in photosynthetic production, and the death decomposition of the understory herb community (Qiu et al., 2020; Lull et al., 2020).

In summary, M, CI and O and soil ST, SM, SBD, SP, SOC, AP, and AN were essential factors affecting the shrub and herb community structure. These factors affect the community structure of herbs in various direct and indirect ways. Among these, the stand spatial structures CI and O were the most fundamental driving factors.

### 4.5 Effects of mixed afforestation on the survival strategies of understory plants

Mixed planting leads to the differentiation of the stand environment, and the understory plant community changes in species composition and quantity proportion through environmental selection (Gong et al., 2021b), reflecting that the composition of ecological niches for resource utilization of the entire community has also changed (Jin et al., 2018). We found that the niche width of the understory shrub community in MCLMF changed significantly compared with MPF. Additionally, the proportion of species with a high niche width increased significantly. There are two reasons for this. First, the community has expanded and settled species, such as *Cunninghamia lanceolata* and *Itea chinensis*. Second, some species with medium niche widths, such as *Camphora officinarum*, *M. japonica*, and *Smilax china*, gradually generalized.

Regarding resource overlap, the proportion of species with low resources in the understory shrub community of MCLMF decreased. In contrast, the proportion of species with high resource overlap (0.6–1) increased significantly. This result shows that MCLMF with better light and heat resources had improved resource sharing, so a few species did not monopolize all survival resources (Fletcher et al., 2012; Sterck et al., 2013). The niche width and resource overlap of the understory shrub community in MLMF did not change significantly compared with the MPF. This result indicated that the MLMF did not improve the resource acquisition of understory shrubs, even though this article found that MLMF increased the diversity of shrubs.

Regarding the herb community, the intermediate niche width of MCLMF and MLMF increased compared with MPF. However, MCLMF is many species with a low niche width decreased, while MLMF is many species with a high niche width decreased. This result indicated that the understory herbs of MCLMF were moving toward generalist species after mixed planting, while the herb community of MLMF was moving toward specialist species. This may be due to the different spatial competition levels and spatial openness of the tree layer. The more intense the spatial competition in the tree layer, the less the utilization of environmental resources, which was beneficial to the survival of understory plants (Prévosto et al., 2016). The more open space, the more light and heat resources the understory herbs could use. Therefore, the understory plants tended to be generalist species (Holmgren and Poorter, 2007). In summary, we found that mixed planting changes the community structure of understory plants and the ecological niche of shrubs and herbs for resource utilization changes. Among them, the niche width of understory shrubs and herbs MCLMF increased, and overall resource utilization was improved.

## 5 Conclusions

Our findings suggest that MCLMF enhanced the diversity of understory shrubs and herbs, increased the proportion of species with high niche width and resource utilization overlap, and promoted understory shrubs and herbs to become generalist species. On the other hand, MLMF enhanced the diversity and niche width of understory shrubs but reduced the niche width and resource utilization overlap of herb species, leading understory herbs to become specialist species. Comprehensively, the M, CI, and O spatial structures of the tree layer were the main factors regulating the understory environment and driving the community structure of the understory plants. Meanwhile, soil ST, SM, SOC, pH, AN, AP, and AK were intermediary factors between the upper stand spatial structure and the understory plant community structure. In conclusion, mixed forests can promote artificial plantation quality and maintaining forests ecosystem sustainable development in fragile ecosystems along rivers and other diversity decline areas by regulating stand structure and enriching plant diversity. In addition, the regulation for O and CI of stand spatial structure should be emphasized to increase plant diversity and improve survival strategies of understory plants.

## Data Availability

The original contributions presented in the study are included in the article/**Supplementary Material**. Further inquiries can be directed to the corresponding author.
