# Supplementary material for: Stand structure influences understory plant diversity through soil factors: three afforestation types of Masson’s pine in the upper Yangtze River, China
Source: Front Plant Sci. 2025 May 30;16:1513038. doi: 10.3389/fpls.2025.1513038 (PMC12162520; doi:10.3389/fpls.2025.1513038)
Supplement: Supplementary file 1 [file DataSheet1.docx]

Supplementary materials:


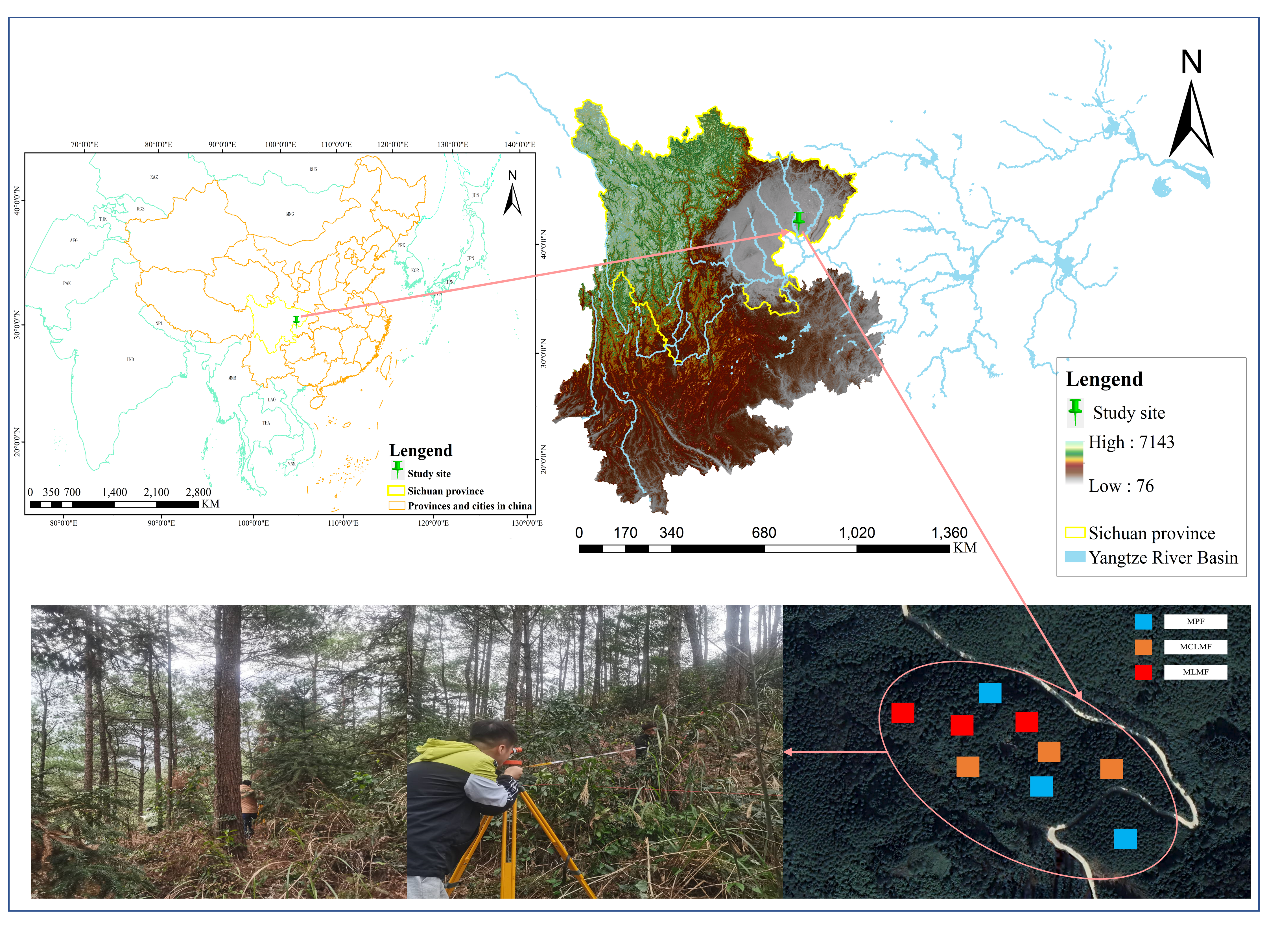


**Fig.S1.** Outline of the research site (Guang 'an City, Sichuan Province, China). The afforestation types are MPF, pure *Pinus massoniana* forest; MCLMF, *Pinus massoniana*–*Cunninghamia lanceolata* mixed forest; MLMF, *Pinus massoniana*–*Liquidambar formosana* mixed forest.


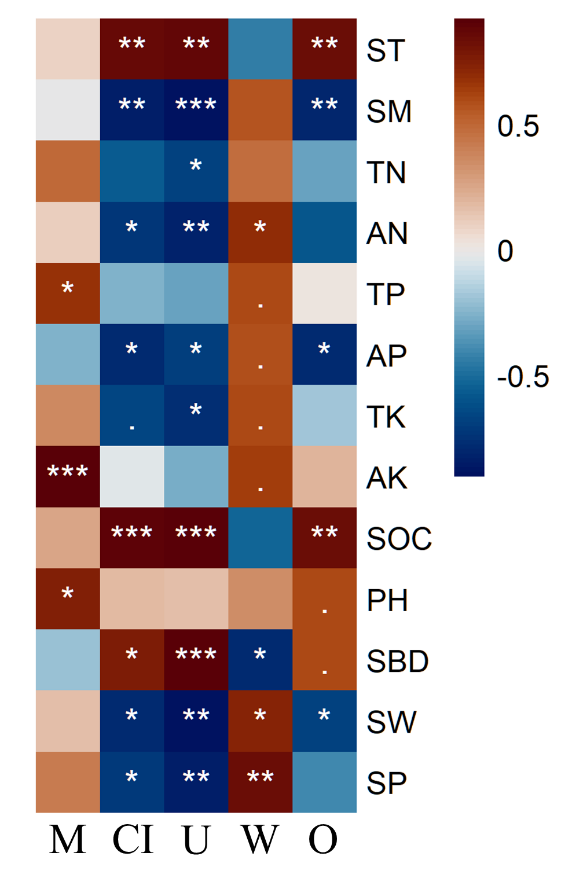


**Fig.S2.** The correlation coefficients between the stand spatial structure characteristic indexes and environmental factors.

Note: The color gradient in the legend ranges from deep blue to white to deep red, representing correlation coefficients from -1 to 1. The color of each grid reflects the degree of correlation between the variable on the X-axis and the variable on the Y-axis. A white asterisk (*) in the grid indicates significance. A single asterisk (*) denotes significance at *p* < 0.05, with an absolute correlation coefficient r greater than 0.7. Two asterisks (**) denote significance at *p* < 0.01, with r > 0.8. Three asterisks (***) denote significance at *p* < 0.001, with r > 0.9. A single white dot (.) indicates an absolute correlation coefficient r greater than 0.6. Grids without any symbols indicate an absolute correlation coefficient r less than 0.6 and non-significant correlation. The stand spatial structure parameters are M, mingling index; CI, competition index; U, neighborhood comparison; W, uniform angle index; O, opening degree. The soil parameters are SOC, soil organic carbon; TN, soil total nitrogen; AN, soil available nitrogen; TP, soil total phosphorus; AP, soil available phosphorus; TK, soil total potassium; AK, soil available potassium; SW, soil water content; pH, soil pH; SBD, soil bulk density; SP, soil porosity.


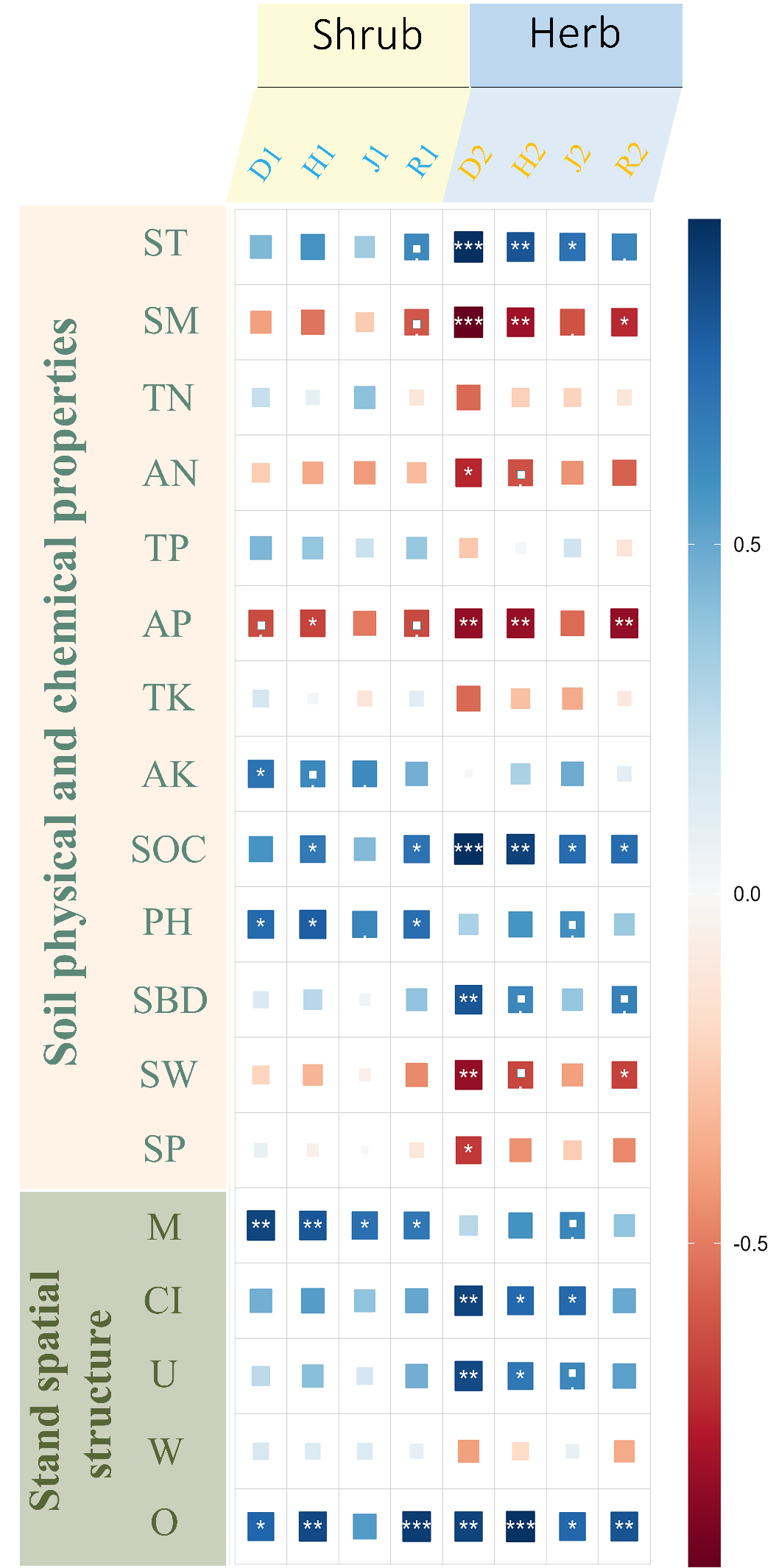


**Fig.S3.** Correlation coefficients between the stand spatial structure characteristic indexes and environmental factors and the diversity of understory plants.

Note: The color gradient in the legend ranges from deep red to white to deep blue, representing correlation coefficients from -1 to 1. The color of each grid reflects the degree of correlation between the variable on the X-axis and the variable on the Y-axis. A white asterisk (*) in the grid indicates significance. A single asterisk (*) denotes significance at *p* < 0.05, with an absolute correlation coefficient r greater than 0.7. Two asterisks (**) denote significance at *p* < 0.01, with r > 0.8. Three asterisks (***) denote significance at *p* < 0.001, with r > 0.9. A single white dot (.) indicates an absolute correlation coefficient r greater than 0.6. Grids without any symbols indicate an absolute correlation coefficient r less than 0.6 and non-significant correlation. The stand spatial structure parameters are M, mingling index; CI, competition index; U, neighborhood comparison; W, uniform angle index; O, opening degree. The soil parameters are ST, soil temperature; SM, soil moisture; SBD, soil bulk density; SW, soil water content; SP, soil porosity; TN, soil total nitrogen; AN, soil available nitrogen; TP, soil total phosphorus; AP, soil available phosphorus; TK, soil total potassium; AK, soil available potassium; SOC, soil organic carbon; pH, soil pH. The diversity parameters are D1-2, simpson index; H1-2,shannon-wiener index; J1-2, pielou index and R1-2, richness index, number 1 and 2 represent shrub communities and herbaceous communities, respectively.

**Table S1.** The basic information of sample plots.

| Basic Indexes | (MPF)  *pinus massoniana* pure forest | (MCLMF)  Mixed forest of *Pinus massoniana* and *Cunninghamia lanceolata* | (MLMF)  *Pinus massoniana* and *Liquidambar formosana* mixed forest |
| --- | --- | --- | --- |
| Slope position | middle | middle | middle |
| Elevation (m) | 654±1.41 | 605.66±6.12 | 642.33±4.98 |
| Density (plants hm^−2^) | 625±154.11 | 675±81.64 | 492±71.26 |
| Average DBH (cm) | 19.63±0.95 | 19.39±1.13 | 24.02±1.34 |
| Canopy density | 0.6 | 0.5 | 0.9 |

**Table S2.** The importance value (IV/%) and niche breadth (Bi) and niche overlap (Oi) of the shrub layer in three different types of stands. MPF (pure *Pinus massoniana* forest), MCLMF (*Pinus massoniana*-*Cunninghamia* *lanceolata* mixed forest), MLMF (*Pinus massoniana*-*Liquidambar formosana* mixed forest).

| Species | Treatment | | | | | | | | |
| --- | --- | --- | --- | --- | --- | --- | --- | --- | --- |
|  | MPF | | | MCLMF | | | MLMF | | |
|  | IV | Bi | OI | IV | Bi | OI | IV | Bi | OI |
| *Camphora officinarum* | 9% | 0.53 | 0.39 | 9% | 1.07 | 0.69 | - | - | - |
| *Maesa japonica* | 10% | 0.55 | 0.40 | 9% | 1.01 | 0.63 | 13% | 1.03 | 0.67 |
| *Quercus × leana* | 3% | 0.69 | 0.54 | - | - | - | - | - | - |
| *Rubus buergeri* | 17% | 1.05 | 0.62 | 12% | 1.00 | 0.69 | 20% | 1.10 | 0.69 |
| *Eurya japonica* | 11% | 1.08 | 0.69 | 5% | 1.01 | 0.63 | 6% | 0.92 | 0.55 |
| *Litsea cubeba* | 4% | 0.63 | 0.54 | 7% | 0.60 | 0.47 | - | - | - |
| *Rubus corchorifolius* | 18% | 0.93 | 0.65 | 8% | 1.01 | 0.63 | 2% | 0 | 0.45 |
| *Smilax china* | 6% | 0.67 | 0.47 | 11% | 1.09 | 0.73 | - | - | - |
| *Mahonia fortunei* | 2% | 0 | 0.44 | - | - | - | - | - | - |
| *Ficus gasparriniana* | 3% | 0 | 0.44 | 5% | 0.98 | 0.72 | 4% | 0.68 | 0.50 |
| *Gardenia jasminoides* | 2% | 0 | 0.44 | 2% | 0.69 | 0.63 | 2% | 0 | 0.33 |
| *Loropetalum chinense* | 4% | 0 | 0.44 | - | - | - | - | - | - |
| *Camellia sinensis* | 4% | 0 | 0.38 | 2% | 0 | 0.35 | 1% | 0 | 0.37 |
| *Synotis nagensium* | 1% | 0 | 0.38 | 2% | 0 | 0.53 | 9% | 0.69 | 0.55 |
| *Rubus* | 4% | 0 | 0.38 | - | - | - | - | - | - |
| *Myrsine africana L.* | 2% | 0 | 0.38 | 4% | 1.05 | 0.68 | 7% | 1.05 | 0.64 |
| *Aralia elata* | - | - | - | 2% | 0 | 0.53 | 1% | 0 | 0.45 |
| *Elaeagnus umbellata* | - | - | - | 2% | 0.64 | 0.64 | 2% | 0.59 | 0.48 |
| *Clerodendrum cyrtophyllum* | - | - | - | 1% | 0 | 0.53 | 1% | 0 | 0.45 |
| *Rubus lambertianus* | - | - | - | 1% | 0 | 0.53 | - | - | - |
| *Artemisia argyi* | - | - | - | 1% | 0 | 0.53 | - | - | - |
| *Oreocnide frutescens* | - | - | - | 1% | 0 | 0.53 | 2% | 0 | 0.45 |
| *Pinus massoniana* | - | - | - | 2% | 0.67 | 0.65 | - | - | - |
| *Itea chinensis* | - | - | - | 3% | 1.02 | 0.72 | 1% | 0 | 0.37 |
| *Serissa japonica* | - | - | - | 2% | 0.66 | 0.64 | - | - | - |
| *Hedera nepalensis var. sinensis* | - | - | - | 1% | 0 | 0.53 | - | - | - |
| *Dalbergia hancei* | - | - | - | 2% | 0 | 0.34 | - | - | - |
| *Cunninghamia lanceolata* | - | - | - | 2% | 0.69 | 0.51 | - | - | - |
| *Pericampylus glaucus* | - | - | - | 2% | 0 | 0.34 | - | - | - |
| *Dalbergia hupeana* | - | - | - | 1% | 0 | 0.35 | - | - | - |
| *Robinia pseudoacacia* | - | - | - | 1% | 0 | 0.35 | - | - | - |
| *Paris polyphylla* | - | - | - | - | - | - | 2% | 0 | 0.36 |
| *Boehmeria nivea* | - | - | - | - | - | - | 2% | 0.69 | 0.60 |
| *Millettia oosperma* | - | - | - | - | - | - | 13% | 0.90 | 0.56 |
| *Sageretia thea* | - | - | - | - | - | - | 4% | 0.64 | 0.48 |
| *Rosa cymosa* | - | - | - | - | - | - | 2% | 0.66 | 0.57 |
| *Viburnum dilatatum* | - | - | - | - | - | - | 3% | 0.69 | 0.59 |
| *Eleutherococcus trifoliatus* | - | - | - | - | - | - | 3% | 0 | 0.47 |
| *Rubus delavayi* | - | - | - | - | - | - | 1% | 0 | 0.47 |

**Table S3.** The importance value (IV/%) and niche breadth (Bi) and niche overlap (Oi) of the herb layer in three different types of stands. MPF (pure *Pinus massoniana* forest), MCLMF (*Pinus massoniana*-*Cunninghamia lanceolata* mixed forest), MLMF (*Pinus massoniana*-*Liquidambar formosana* mixed forest).

| Species | Treatment | | | | | | | | |
| --- | --- | --- | --- | --- | --- | --- | --- | --- | --- |
|  | MPF | | | MCLMF | | | MLMF | | |
|  | IV | Bi | OI | IV | Bi | OI | IV | Bi | OI |
| *Dicranopteris pedata* | 41% | 1.09 | 0.70 | 20% | 0.68 | 0.50 | - | - | - |
| *Miscanthus sinensis* | 13% | 1.03 | 0.70 | 15% | 1.08 | 0.69 | - | - | - |
| *Pteridium aquilinum var. latiusculum* | 22% | 1.06 | 0.68 | 21% | 1.09 | 0.72 | 22% | 1.03 | 0.67 |
| *Dryopteris Adanson* | 5% | 0 | 0.36 | - | - | - | - | - | - |
| *Loropetalum chinense* | 1% | 0 | 0.36 | - | - | - | - | - | - |
| *Pericampylus glaucus* | 11% | 1.02 | 0.64 | 5% | 0.64 | 0.48 | 4% | 0.68 | 0.55 |
| *Synotis nagensium* | 3% | 0.69 | 0.51 | - | - | - | - | - | - |
| *Liriope spicata* | 1% | 0 | 0.31 | 2% | 0.68 | 0.59 | 1% | 0 | 0.42 |
| *Setaria palmifolia* | 1% | 0 | 0.44 | 4% | 0.61 | 0.59 | 7% | 0.67 | 0.48 |
| *Oplismenus compositus* | 1% | 0 | 0.44 | - | - | - | - | - | - |
| *Clematoclethra lasioclada* | 1% | 0 | 0.44 | - | - | - | - | - | - |
| *Iris japonica* | - | - | - | 8% | 0 | 0.47 | 42% | 1.07 | 0.64 |
| *Rubia alata* | - | - | - | 1% | 0 | 0.47 | - | - | - |
| *Tetrastigma hemsleyanum* | - | - | - | 8% | 1.04 | 0.70 | 3% | 0 | 0.35 |
| *Carex baccans* | - | - | - | 3% | 0 | 0.47 | 8% | 0.46 | 0.42 |
| *Lophatherum gracile* | - | - | - | 3% | 0.69 | 0.61 | - | - | - |
| *Aster tataricus* | - | - | - | 4% | - | 0.35 | - | - | - |
| *Semiaquilegia adoxoides* | - | - | - | 3% | - | 0.33 | - | - | - |
| *Lysimachia christinae* | - | - | - | 2% | 0 | 0.33 | 2% | 0 | 0.42 |
| *Arachniodes rhomboidea* | - | - | - | - | - | - | 2% | 0 | 0.35 |
| *Corydalis pallida* | - | - | - | - | - | - | 3% | 0 | 0.29 |
| *Lygodium japonicum* | - | - | - | - | - | - | 3% | 0.69 | 0.53 |
| *Corydalis sheareri* | - | - | - | - | - | - | 5% | 0 | 0.42 |
